# Molecular specializations of deep cortical layer analogs in songbirds

**DOI:** 10.1038/s41598-020-75773-4

**Published:** 2020-10-30

**Authors:** Alexander A. Nevue, Peter V. Lovell, Morgan Wirthlin, Claudio V. Mello

**Affiliations:** Department of Behavioral Neuroscience, Oregon Health and Science University, Portland, OR USA

**Keywords:** Neuroscience, Molecular neuroscience, Motor control, Sensorimotor processing

## Abstract

How the evolution of complex behavioral traits is associated with the emergence of novel brain pathways is largely unknown. Songbirds, like humans, learn vocalizations via tutor imitation and possess a specialized brain circuitry to support this behavior. In a comprehensive in situ hybridization effort, we show that the zebra finch vocal robust nucleus of the arcopallium (RA) shares numerous markers (e.g. *SNCA*, *PVALB*) with the adjacent dorsal intermediate arcopallium (AId), an avian analog of mammalian deep cortical layers with involvement in motor function. We also identify markers truly unique to RA and thus likely linked to modulation of vocal motor function (e.g. *KCNC1, GABRE*), including a subset of the known shared markers between RA and human laryngeal motor cortex (e.g. *SLIT1*, *RTN4R*, *LINGO1*, *PLXNC1*). The data provide novel insights into molecular features unique to vocal learning circuits, and lend support for the motor theory for vocal learning origin.

## Introduction

An in-depth understanding of how the brain controls learned behaviors and how these behaviors arise in specific animal lineages requires detailed knowledge of the molecular organization of the underlying circuits. Songbirds offer an excellent model for investigating these questions. Their vocal circuitry has been extensively studied, and consists of interconnected pallial, basal ganglia, and thalamic components that control the production and acquisition of learned vocalizations^1^. As is typical of birds, the pallial (cortical-like) areas consist of discrete nuclei, in contrast to the layered cortex of mammals^2–4^. The songbird vocal circuitry can be subdivided into a direct vocal-motor pathway, necessary for song production, and an anterior pathway, involved in vocal learning and adult vocal plasticity^5–8^. Discrete nuclei of both pathways have also been identified in the other vocal learning avian groups (i.e. parrots^9–11^ and hummingbirds^12,13^) but are absent or rudimentary in vocal non-learning birds^13–18^. Notably, vocal learning behavior provides an important basis for spoken language in humans^19,20^ and the related circuits in vocal learning birds and humans share remarkable convergent molecular specializations^21^. In contrast, with the possible exception of a few other mammalian groups such as bats and cetaceans^22,23^, the occurrence of vocal learning and related circuitry is quite rare among vertebrates, and seems absent or only rudimentary in rodents and non-human primates^24–28^.

Despite considerable knowledge on the anatomical, physiological, and molecular properties of the songbird vocal circuitry, especially in zebra finches, our understanding of the evolution of these anatomically and functionally distinct vocal nuclei is limited. The close proximity of the vocal nuclei to auditory areas has led to the hypothesis that these circuits may have evolved from circuits involved in song perceptual processing^29–31^. Alternatively, based on their close proximity to areas thought to be involved in motor control, it has been proposed that vocal nuclei evolved as specialized expansions of preexisting motor regions^32–34^. Progress towards testing these hypotheses has remained limited, however, possibly because most effort has focused on characterizing the unique properties of the vocal circuitry rather than on how they relate to other brain areas.

The robust nucleus of the arcopallium (RA) is a particularly prominent and extensively studied vocal nucleus in zebra finches. It is the major forebrain vocal output nucleus and is thought to encode important acoustic features of finch song motifs^35–37^. The RA is considered part of the intermediate arcopallium, which is the major source of descending output from the avian telencephalon^38^. The arcopallium, more broadly, is thought to contain the avian analog of the deep layers of the mammalian sensory and motor cortices based on similarities in their projection patterns^31,39^, neuronal activation^33,40^, and transcriptional profiles^41–44^, but may also contain the avian equivalent of pallial parts of the mammalian amygdala^44–46^. Avian analogs of RA are found in other birds that evolved vocal learning^9–13^, but are thought to be absent in vocal non-learning birds based on cytoarchitectonics and molecular criteria^14–16,47^; but see also^17,18^. Transcriptomics studies^21,48,49^ have identified several hundred differentially expressed genes in RA compared to the adjacent ventral intermediate arcopallium. Of these, a subset were described as molecular specializations shared with the analogous nuclei in other vocal learner birds and the laryngeal representation of the primary motor cortex (LMC) in humans^21^, suggesting that a shared gene network may have convergently evolved across different vocal learning systems. Notably, however, an extensive examination of the brain expression patterns of shared RA and LMC markers has not yet been performed.

We have previously described the differential expression of a small set of genes in both RA and the adjacent nucleus the dorsal intermediate arcopallium (AId; referred to as LAI, Ad, and AI in previous studies), as well as the sharp borders of *SCN3B* expression for both RA and AId^44^. These observations are consistent with previous indications of similar connectivity between RA and AId. For example, RA and AId receive parallel input from the nidopallium^6,50,51^ and also send distinct but parallel projections to the brainstem that can be considered analogous to those in the cortico-bulbar tract in mammals^39,51^. Other studies suggest common motor control functions of RA and AId. For example, AId shows immediate early gene expression after movements such as wing flapping or hopping^33,40^, analogous to RA being active during song production^35,36,52^. Furthermore, while lesions to RA result in severe song deficits^5^, birds show marked motor deficits including akinesia and immobility in large lesions that primarily include AId^53^. These observations are consistent with AId being involved in somatic motor control^33^, though other studies suggest a role in vocal learning^54^. It has been previously suggested that AId might be broadly present in birds, regardless of vocal learning, and that RA may have originated as an expansion and specialization of AId^33,44,55^. Nonetheless, AId remains poorly defined, and our knowledge of its molecular organization is limited. A closer comparison of gene expression patters in RA and AId is also needed to more clearly identify features unique to the vocal circuitry.

Our main goals were to improve our understanding of the molecular organization of AId in comparison with RA, and to better define molecular properties unique to RA. Using in situ hybridization for markers with sharp expression boundaries, we first generated a more precise definition of AId in adult male zebra finches. We then conducted extensive analysis to distinguish molecular features common to RA and AId from those unique to RA. We also were able to identify AId in pre-song juvenile males and non-singing females, as well as in two suboscine species, a sister taxa to songbirds generally thought to lack vocal learning and/or related forebrain vocal nuclei^16^. Our data provide substantial further support for a close molecular similarity between RA and AId, as well as a more in-depth definition of features unique to RA and the vocal control circuitry. They suggest that AId may represent a subdivision of the arcopallium that existed prior to the emergence of vocal learning circuits in birds, and are consistent with the hypothesis that RA may have evolved as a specialization of AId.

## Methods

### Animals and tissue preparation

All procedures involving live animals were approved by the OHSU Institutional Animal Care and Use Committee and are in accordance with NIH guidelines. Adult (n = 11) and fully fledged 20-day post-hatch (dph) (n = 2) male, and adult female (n = 2) zebra finches (*Taeniopygia guttata*) were obtained from our colony or purchased from a local breeder. Adult finches were isolated in sound dampening chambers overnight and sacrificed by decapitation the next morning prior to lights on to minimize the potential confounds of singing and auditory stimulation on activity dependent changes in gene expression. To minimize possible adverse effects of stress, juvenile males were not sound isolated, and instead were removed directly from the aviary and sacrificed by decapitation a few minutes after lights on. Juvenile males could usually be identified by plumage, however we also confirmed sex by gonadal inspection. For all birds, immediately after sacrifice, their brains were dissected and blocked in either the sagittal plane (n = 3 adult male brains), or in the frontal plane (all other brains) at a level just rostral to the tectum, and frozen in Tissue-tek (Sakura-Finetek) in a dry ice/isopropyl alcohol slurry. Brains were sectioned on a Leica CM1850 cryostat at 10 μm thickness and mounted on charged microscope slides (Superfrost plus; Fisher Scientific). Sections were post fixed for 5 min at room temperature in a solution containing 3% paraformaldehyde in phosphate buffered saline (PBS), washed twice in PBS, dehydrated in an ethanol series, and stored at − 80 °C until use.

We also processed brains from a Willis’s antbird (n = 1 male; *Cercomacroides laeta*) and a Straight-billed woodcreeper (n = 1 male; *Dendroplex picus*). These birds had been captured at field sites in the suburbs of Belém (Pará, Brazil) and deposited at the Emilio Goeldi Museum (Belém, PA, Brazil). Shortly after euthanasia, the brains were frozen and stored at − 80 °C. These cryopreserved museum samples were subsequently processed in the frontal plane as described above for zebra finches.

### In situ hybridization

We generated in situ hybridization data for 61 genes, including 46 genes previously identified as shared markers between avian RA analogs and human LMC^21^ and/or highly differential markers of RA in zebra finches^49^. For each gene examined, we initially confirmed orthology between zebra finch and other clades by a combination of cross-species alignments and synteny verification with other birds (e.g. chicken) and bird outgroups (e.g. mouse, human), and non-avian sauropsids (e.g. anole), using UCSC’s genome browser and the BLAT toolkit as previously detailed^4^. We then identified appropriate clones from the ESTIMA brain EST/cDNA library^56^ for riboprobe synthesis. To maximize specificity, whenever possible we avoided clones containing protein-coding regions and selected those containing only or primarily the 3′-untranslated sequence. The clones selected for each gene were confirmed to align significantly to a single locus in the zebra finch genome. The clones for 36 of the genes included in the present study are listed in Supplemental Table 1; clone details for the remaining genes examined in the present study can be found in the Zebra finch Expression Brain Atlas (ZEBrA) website (http://www.zebrafinchatlas.org). Further details on our criteria and pipeline for clone selection for in situs has been previously described^4^.

**Table 1:** Classification of RA markers based on in situ patterns.

	RA unique markers	RA and AId markers
Shared human LMC markers	C1QL3, CNTN3, CYGB, DAAM1, DGKZ, DPYSL3, GABRB3, GPRC5B, KCTD15, LINGO1, MTCL1, NEUROD6, NEXMIF, NTRK2, RORA, RTN4R, SAP30, SLIT1, SMAP1, UCHL1, YWHAH	B3GAT1, CDH4, CDH11, DOCK4, ETNK1, FAM49A, GAP43, GNG2, GPM6A, LYPD1, NECAB2, NOL4, PCDH17, PLXNC1, PPFIA2, PPP2R5C, PVALB, SLC25A22, SNCA, SYNPR, SYT17, TENM3, TMEFF2, VIP, ZBTB18
Other markers	ABCG4, ACKR3, ADCYAP1, ARPC5, B3GNT2, BTBD10, CAMK2A, CAMKK1, CDH8, CHRM4, CRHR2, CTSL, DPP6, GABRE, GRK3, HTR1B, IGF1, IL1RAPL2, KCNA6, KCNC1, KCNF1, KCNJ6, KCNK12, KCNS2, KCNT2, KCTD20, KIF26B, LRP8, MAP4, MGAT4C, NTNG1, P4HA2, PLXNA4, PTPRN2, PTPRU, RELN, RHOB, RIMS4, SCN2A, SEPT12, SEPT6, SH3BP5, SLC8A1, STK26, STMN1, SYBU, TNFAIP8L3	ADAM23, ADRA1D, ANXA6, APOH, ARHGDIB, ATP2A3, ATP2B2, BAIAP2, CABP1, CACNA1E, CACNA2D2, CACNB2, CAMTA1, CD99L2, CDH9, CNTN4, CNTNAP2, CPNE2, CRHR1, DHCR24, FAM163B, FLRT2, GDA, GLRA2, GLRA4, GRIN2B, GRM3, HTR2A, HRH3L, JPT1, KCNAB1, KCNAB2, KCNS1, KCTD12, KCTD16, KCTD3, LPL, MPZL1, , NEFL, NEFM, NEGR1, NRXN1, NTSR1, PAK6, PCP4, PLD1, PLS3, PLXNA1, PRKAR1B, PTPRZ1, QSOX1, RCAN2, RGS4, ROBO1, SAP30L, SCN1A, SCN3B, SCN4B, SCN8A, SCUBE1, SEMA7A, SLC24A2, SLC4A4, SLC6A7, SYF2, SYNGR3, UGT8, UNC5D, WASF1

Genes differentially expressed in RA only (RA unique markers) are separated from genes that are differential markers of both areas (RA and AId markers). For both groups, we further indicate whether RA markers were previously reported or not as shared markers with human LMC^21^.

We followed protocols for riboprobe synthesis and purification, and in situ hybridization as described previously^57^. Briefly, selected cDNA clones were grown overnight, and plasmids were isolated (QIAprep Spin Miniprep Kit), digested with BssHII, and purified (QIAquick PCR purification kit). Digoxygenin(DIG)-labeled antisense riboprobes were then synthesized using T3 RNA polymerase (Promega) and a DIG RNA labeling mix (Roche) for 2 h at 37 °C. Riboprobes were purified using Sephadex G-50 columns and stored at 20 °C until use. Incubations with a no probe negative control, or positive control probe for a gene with a known expression pattern (e.g. GAD2) were routinely included in hybridizations.

Prior to hybridization, slides were acetylated for 10 min in a solution containing 1.35% triethanolamine and 0.25% acetic anhydride. Slides were briefly washed in 2X SSPE (300 mM NaCl, 20 mM NaH_2_PO_4_-H_2_O) and dehydrated in an ethanol series. A hybridization solution consisting of 50% formamide, 2X SSPE, 2 μg/μL tRNA, 1 μg/μL BSA, 1 μg/μL Poly A, and 2 μL DIG-labeled riboprobe in DEPC-treated H_2_O was prepared. Slides were coverslipped and hybridized in a mineral oil bath overnight at 65 °C. The next day, the slides were washed in chloroform to remove the mineral oil and washed in SSPE to remove the coverslips. Slides were then washed in 50% formamide 2X SSPE solution followed by two 30 min washes in 0.1X SSPE at 65 °C, agitated every 10 min. Following the high stringency washes, the sections were briefly permeabilized in TNT (100 mM Tris–HCl pH 7.4, 150 mM NaCl, 0.3% Triton X-100). Slides were then blocked in TNB (100 mM Tris–HCl pH 7.4, 150 mM NaCl, 0.36% w/v BSA, 1% skim milk) for 30 min in a humidified chamber at room temperature. Slides were then incubated in an alkaline phosphatase conjugated anti-DIG antibody (Roche, 1:600) in TNB for 2 h in a humidified chamber at room temperature. Slides were then washed twice for 15 min in TMN (100 mM Tris–HCl, 150 mM NaCl, 5 mM MgCl_2_) and incubated for 1–3 days in filtered BCIP/NBT Substrate Solution (PerkinElmer) at room temperature. After incubation, slides were rinsed in DI water, fixed in 3% paraformaldehyde, and washed again in DI water. Slides were then coverslipped with VectaMount permanent mounting medium (Vector).

All genes for which we generated in situ hybridization data were assessed in at least two brains of adult male zebra finches. Our in situ pipeline, consistent with that described for the ZEBrA database^4^, consisted of an initial assessment of hybridization conditions and general expression pattern in one brain cut in the sagittal plane, and a final hybridization with sections containing RA and AId from another brain. *SCN3B* was run on all brains that were part of the study. For the AId reconstruction, the final hybridizations for *SCN3B* were run in frontal male and female brain series (2 brains each); specifically, every 10th slide (200 µm intervals) in the range that spans the arcopallium was stained for Nissl (cresyl violet), and adjacent slides were processed for *SCN3B *in situ. For the other 60 genes that were examined in frontal sections, effort was made to run the final hybridization at a level around the core region of RA and AId in at least one brain, including both the right and left hemispheres. The remaining 101 genes in this study were assessed in the sagittal plane only, most of them consisting of data that were already available on the ZEBrA website. A complete list of genes, and whether they were assessed in sagittal only or in both sagittal and frontal planes, can be found in Supplemental Table 2. Images from sagittal sections for most of the data generated here are being prepared for uploading to ZEBrA. Lastly, we note that the data in ZEBrA were generated using both left and right hemispheres, thus that database in its current form is not appropriate for evaluating possible hemispheric differences.

### In situ mapping and image analysis

For the AId reconstruction in the male and female frontal brains, the boundaries of major features such as section borders and laminae as seen in the Nissl-stained sections spanning the arcopallium (every 10th slide, 200 µm intervals) were drawn using Neurolucida. In the adjacent hybridized sections, we then drew major section borders and the internal arcopallial boundaries that were defined by the differential expression of *SCN3B*. The resulting drawings were aligned to transverse sections^44^.

For a qualitative analysis of markers of RA and/or AId, we visually examined the in situ patterns of 162 genes, including the patterns in frontal sections generated in this study as well as the available patterns in sagittal sections for another 116 genes classified as RA markers on the ZEBrA website. Our present analysis consisted of comparing gene expression in major arcopallial domains representing subdivisions of the anterior, medial, dorsal, posterior, intermediate, and ventral arcopallium, noting that to simplify the analysis we collapsed the previously defined 19 arcopallial subdivisions^44^ into 12 major domains/subdomains for which we have numerous markers in ZEBrA. Genes that showed expression in a given arcopallial domain similar to the differential expression in RA were considered markers of both RA and that arcopallial domain. The comparison was not exclusive, so RA could share expression of a given marker with multiple arcopallial domains. The same approach was taken for the analysis of AId compared to other arcopallial subdomains. In this case, we started with the list of genes we identified as RA and AId markers (n = 94 genes; see Table 1 in “Results”), noting that all known markers of AId are also markers of RA, as observed in the current study and in previous efforts^44^. For this reason, in the AId comparative gene expression analysis, RA was not included as a subdomain.

For a subset of genes examined in frontal sections (n = 30), we measured relative expression levels within subdivisions of the intermediate arcopallium. This gene subset consisted of genes that were hybridized in closely adjacent sections, thus allowing a consistent evaluation across genes. Included were 7 genes qualitatively classified by visual inspection as markers of RA and AId, as well as 23 genes classified as markers of RA only. Using the FIJI distribution of NIH ImageJ^58^, we performed average optical density measurements in 200 µm × 200 µm windows placed over RA and AId. We then calculated a relative expression ratio using the following ratio: RA_OD_/AId_OD_, where RA_OD_ is the mean 8-bit grayscale value in RA for a given gene, and AId_OD_ is the mean grayscale value in AId for that same gene. Thus, ratio values close to 1 correspond to genes with similar levels of expression in RA and AId, and values deviating from 1 correspond to genes that are differentially expressed in RA, either positive (ratio > 1), or negative (ratio < 1) compared to AId.

### Bioinformatics

We used ConsensusPathDB^59^ to conduct a gene set over-representation analysis to identify individual genes associated with specific biological pathways. Our analysis was conducted using all available pathway databases, a minimum overlap with our input list of two genes and a p-value cutoff of 0.015. We analyzed separately two non-overlapping gene input lists (see Table 1 in “Results”), consisting of genes identified by in situ hybridization analysis and/or examination of ZEBrA patterns as: (1) showing differential expression in both RA and AId (RA and AId markers) and (2) showing differential expression in RA but not AId (RA unique markers). Both positive and negative markers were included in both sets to identify biological pathways under differential regulation. The background consisted of the full set of genes present in the zebra finch Agilent microarray and previously used to define the RA transcriptome^21,48,49^; curation of this oligonucleotide microarray is described in Lovell et al.^60^.

## Results

**Figure 1:**
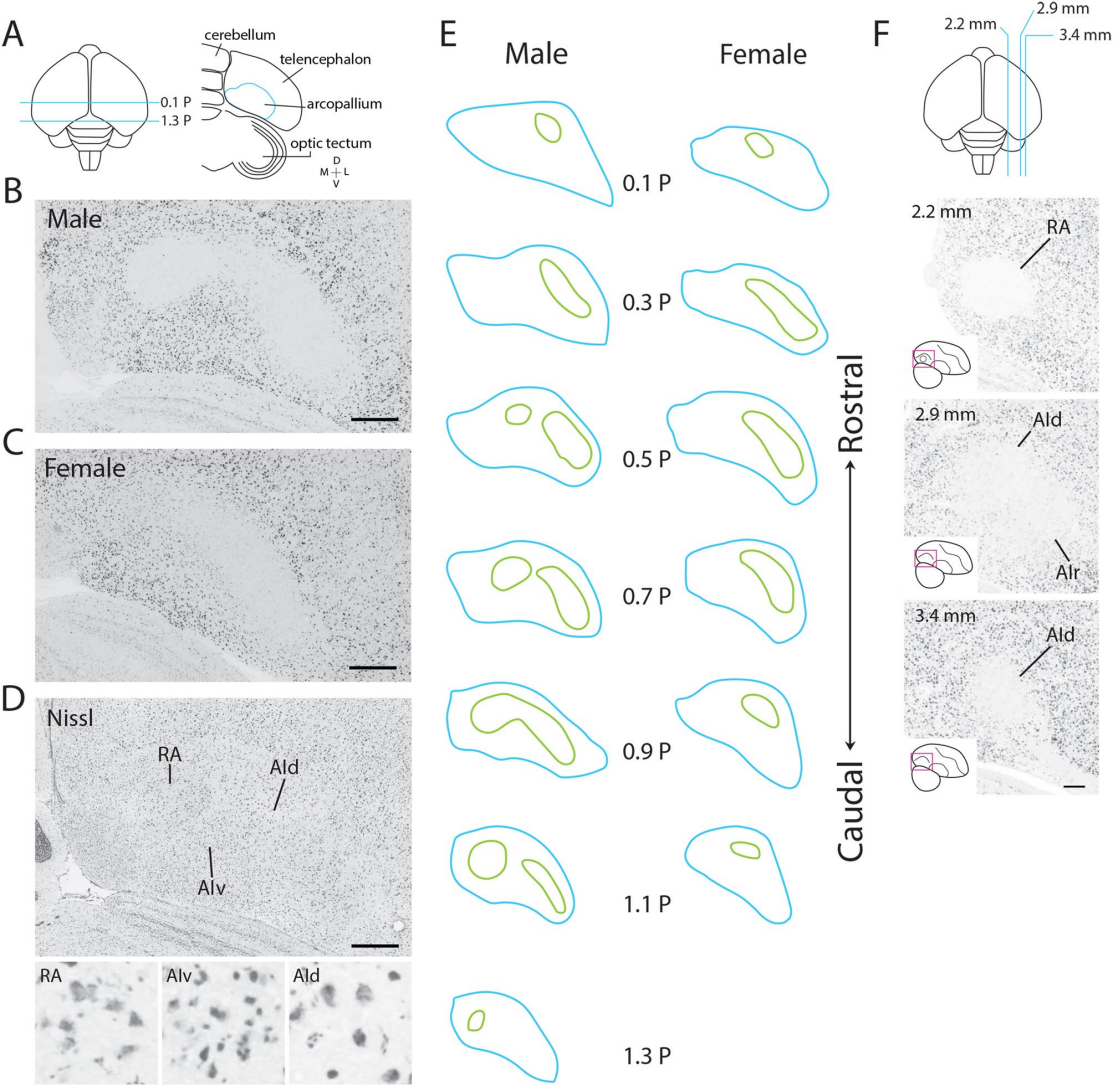
Molecular definition of RA and AId in adult zebra finches. (**A**) Top left: Top-down view of a schematic drawing of the zebra finch brain; blue lines indicate the range of frontal sections examined in this study. Top right: Drawing of a frontal section at 0.7P; blue line indicates the boundaries of the arcopallium, seen under Nissl staining. (**B**,**C**) *SCN3B *in situ hybridization images from a male and a female. RA and AId appear continuous in the male, and RA is indistinguishable in the female. (**D**) Nissl-stained frontal section through arcopallium at the center of RA in a male; RA, but not AId, has clear cytoarchitectonic boundaries. Small panels show high power views (100 × 100 µm images) within RA, AIv, and AId. (**E**) Drawings depicting *SCN3B* expression boundaries (green) in serial frontal sections through the arcopallium (blue) of adult male (left) and female (right) zebra finches. (**F**) Sagittal series of *SCN3B *in situ hybridization images through the arcopallium, reproduced from ZEBrA (www.zebrafinchatlas.org); section level is indicated by the blue lines in the schematic drawing at the top. *AId* dorsal intermediate arcopallium, *AIr* rostral intermediate arcopallium, *AIv* ventral intermediate arcopallium, *RA* robust nucleus of the arcopallium. Scale bar: 400 µm for all images.

To provide complete and precise definitions of the dorsal intermediate arcopallium (AId) and of RA (Fig. 1A), we mapped the expression of *SCN3B* in adult zebra finches using in situ hybridization. In males, the *SCN3B*-defined RA boundaries corresponded closely to cytoarchitectonic boundaries under Nissl (Fig. 1B,D). In contrast, the borders of *SCN3B*-defined AId could not be seen under Nissl, however this region contained neuronal cells with large somata that resemble the projection neurons found in RA, and thus may correspond to AId projection neurons^61,62^. They contrast sharply with the smaller and densely packed cells in the adjacent ventral intermediate arcopallium (AIv; Fig. 1D), or the dorsal arcopallium (AD; not shown). The *SCN3B-*defined AId closely matches the region containing the projection terminals from the shell of the lateral magnocellular nucleus of the anterior nidopallium (LMAN) shell^51^. In females, where RA is atrophied^61,63,64^ an *SCN3B*-defined AId, but not RA, was clearly visible (Fig. 1C). Importantly, both RA and AId were identifiable in adjacent sections via differential *PVALB *in situ, and RA was visible under Nissl staining as a small nucleus with high cell density directly medial to the *SCN3B*-defined AId (Fig. S1). Expression borders were then drawn on serial transverse *SCN3B* sections (200 µm intervals) throughout the whole extent of the arcopallium of adult birds. In males, *SCN3B*-defined AId occupied an extensive area, with a rostro-caudal extent (~ 0.1 P to 1.1 P) somewhat larger than RA (Fig. 1E left). No distinct expression boundary was distinguishable between AId and RA at the core of RA (0.9 P in Fig. 1E left), in fact these two areas formed a medial-to-lateral continuum of low expression. At rostral or caudal levels RA and AId were separated by regions of high *SCN3B* expression (0.5 P or 1.1 P in Fig. 1E left). *SCN3B*-defined AId had a similar location in females (Fig. 1E right) but appeared smaller than in males caudally. In sagittal *SCN3B *in situ images (from ZEBrA), AId was distinguishable from the surrounding arcopallium as a core area of low expression lateral to RA (Fig. 1F) with distinct cytoarchitectonics^4,65^ and continuous with a rostral domain (AIr in Fig. 1F, middle) previously defined in Mello et al.^44^.

**Figure 2:**
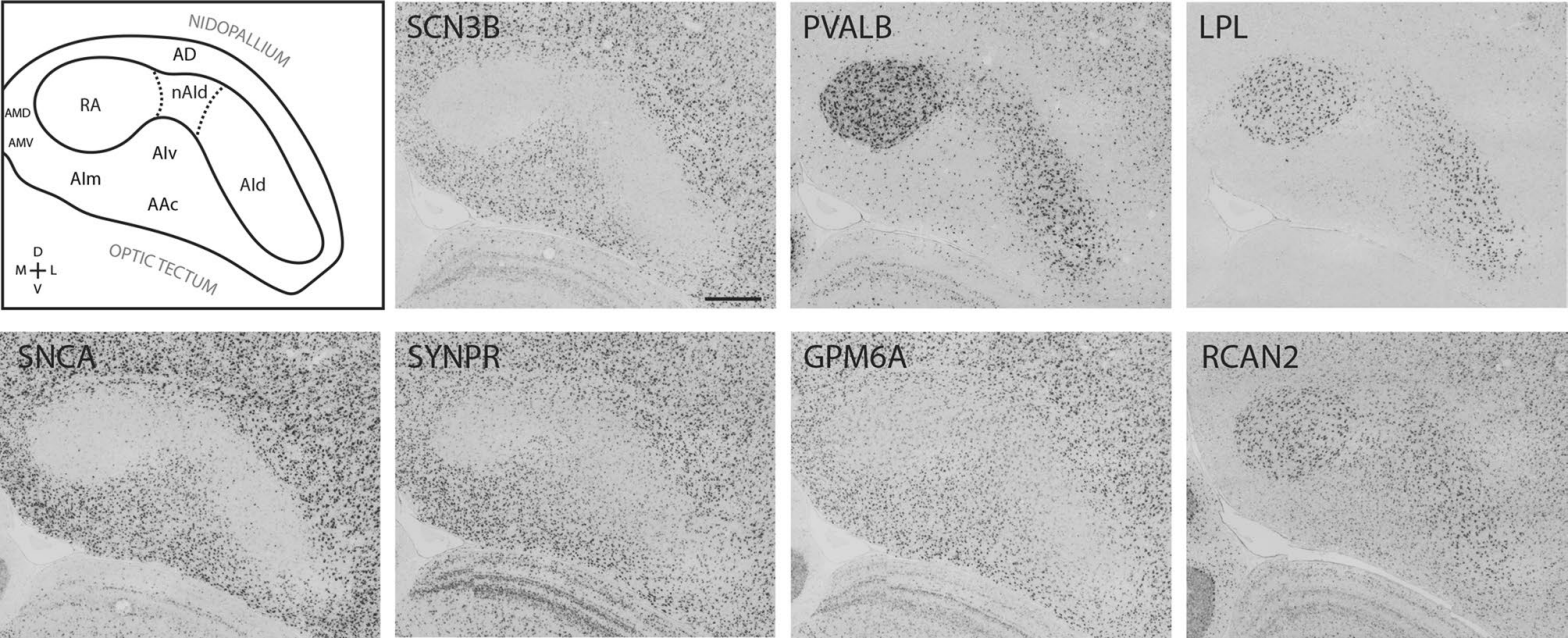
Expression patterns of RA and AId markers. Top left: Drawing of the zebra finch arcopallium in the frontal plane, depicting structures shown in all other panels. RA and AId were defined based on the SCN3B expression pattern in the next panel, placement of other domains derives from fig. 17 in Mello et al.^44^. Other panels: in situ hybridization images for various RA and AId markers. Scale bar: 400 µm for all images. *AAc* caudal anterior arcopallium, *AD* dorsal arcopallium, *AId* dorsal intermediate arcopallium, *AIm* medial intermediate arcopallium, *AIv* ventral intermediate arcopallium, *AMD* dorsal medial arcopallium, *AMV* ventral medial arcopallium, *nAId* neck of the dorsal intermediate arcopallium, *RA* robust nucleus of the arcopallium.

**Figure 3:**
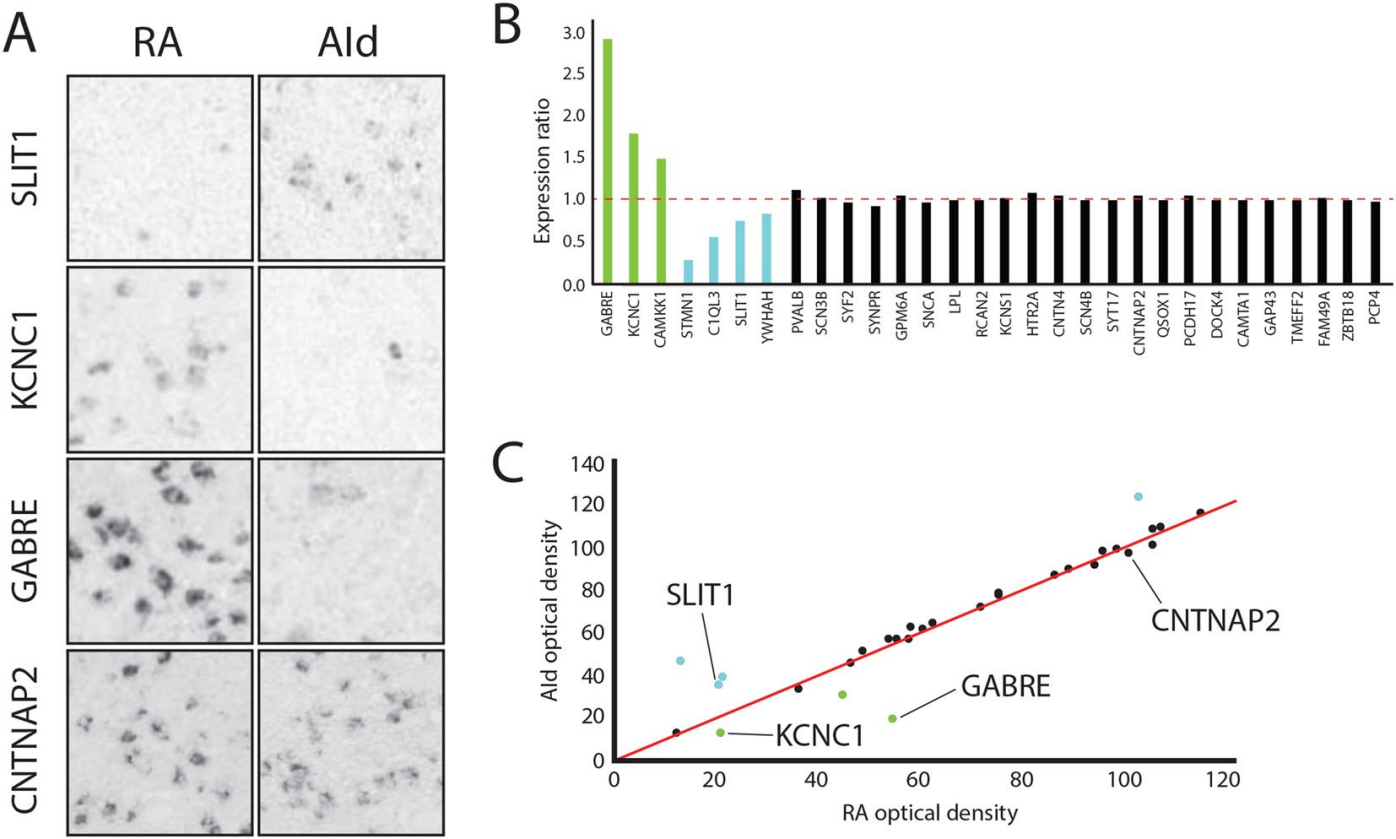
Defining molecular specializations unique to RA or common to both RA and AId. (**A**) High magnification (200 × 200 µm) in situ hybridization images of RA and AId showing cell-level expression of select genes RA unique and RA and AId markers. *SLIT1* (top) and *KCNC1* and *GABRE* (middle) are respectively negative and positive markers unique to RA, whereas expression of *CNTNAP2* (bottom) is similar in RA and AId. (**B**) Expression ratio (optical density within RA/optical density within AId) for genes visually determined to be positive (green) or negative (blue) markers of RA only, or markers of both RA and AId (black). A ratio of 1 (dashed red line) corresponds to a gene equally expressed in RA and AId, and ratios > 1 or < 1 correspond, respectively, to positive or negative RA unique markers. (**C**) A scatterplot of expression ratio values for the genes in (**B**), with RA unique markers deviating from the 1:1 expression ratio line (red).

To better characterize the molecular relationships between AId and RA, we next examined the in situ hybridization patterns of 162 genes that are differentially expressed in RA. This analysis included a set of 46 genes previously identified through microarray screenings as markers that RA in finches (and analogous nuclei in vocal learning birds) share with the laryngeal motor cortex (LMC) in humans^21^ and 116 genes identified as RA markers in ZEBrA^4^. For a set of 60 genes that included the 46 shared RA and LMC marker set and 14 RA markers from ZEBrA, we ran in situ hybridizations in frontal sections containing both RA and AId (examples in Fig. 2), noting that an assessment of the expression in AId had previously not been performed for most of these genes. For the other genes in the RA marker set, we evaluated expression in the sagittal image series available on the ZEBrA website. Among the 162 genes analyzed (full list in Supplemental Table 2), numerous had much lower expression in RA and AId compared to the surrounding arcopallium (e.g. *SCN3B* and *SNCA* in Fig. 2), whereas other genes were more highly expressed in both nuclei compared to the surrounding arcopallium (e.g. *PVALB* and *LPL* in Fig. 2). Yet other genes were more only modestly differential in RA and AId (e.g. *SYNPR*, *GPM6A* and *RCAN2* in Fig. 2), showing regional expression level differences rather than highly differential patterns. Notably, some genes showed less differential expression compared to the surrounds in the dorso-medial part of AId close to the boundary with RA than in the more ventro-lateral AId. We refer to this dorso-medial area as the neck of AId (nAId; top left drawing in Fig. 2), and suggest that it may correspond to a transition zone between RA and AId as previously described based on connectivity^53^. Lastly, numerous other genes were only differentially expressed in RA but not in AId (e.g. KCNC1 and GABRE in Fig. 3). We note that the patterns for the set of genes assessed in frontal sections (Supplemental Table 2) were qualitatively similar in both the left and right hemispheres.

Based on this analysis, we classified each gene as being either a unique marker of RA, which is more likely related to the neurobiology of learned vocalizations, or a marker of both RA and AId, which may represent features of motor control circuits rather than specializations unique to vocal-motor control (Table 1). Notably, of the shared markers of songbird RA and human LMC (n = 46, Pfenning et al.^21^), a large proportion (58%, n = 25) were also differential in AId (Table 1, RA and AId markers), whereas only 42% (n = 21) turned out to be RA unique markers (Table 1, RA unique markers). To provide quantitative support for our visual assessment, we calculated a ratio of expression levels in RA compared to AId for a subset of the genes examined. Genes that were classified as having qualitatively similar levels of expression in RA and AId (Table 1; e.g. *CNTNAP2* in Fig. 3A) were found to have ratios very close to 1 (Fig. 3B, black columns; SD: 0.036), whereas genes classified as unique positive or negative markers of RA (e.g., *KCNC1* and *GABRE*, or *SLIT1* in Fig. 3A, respectively) had expression ratios that were higher (> 1) or lower (< 1) in RA than AId (Fig. 3B, green and blue columns, respectively). Plotting the densitometric ratios for all genes quantified revealed that both RA unique markers as well as RA and AId markers could be found over a wide range of expression levels. These data thus support our visual classification of in situ patterns based on relative differences in expression in RA vs AId.

**Figure 4:**
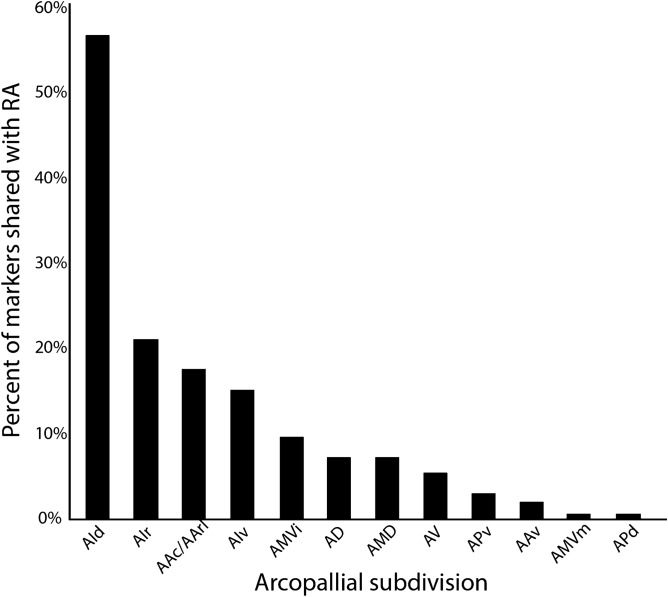
Molecular relationship between RA and other arcopallium domains. The arcopallial expression patterns of 162 RA markers were analyzed based on in situ hybridization data from the present study and from ZEBrA. Plotted are the percentages of RA markers that were also considered markers of other arcopallial domains; individual genes can be represented in multiple columns. Abbreviations: For a complete list of abbreviations see the legend in Fig. 5.

**Figure 5:**
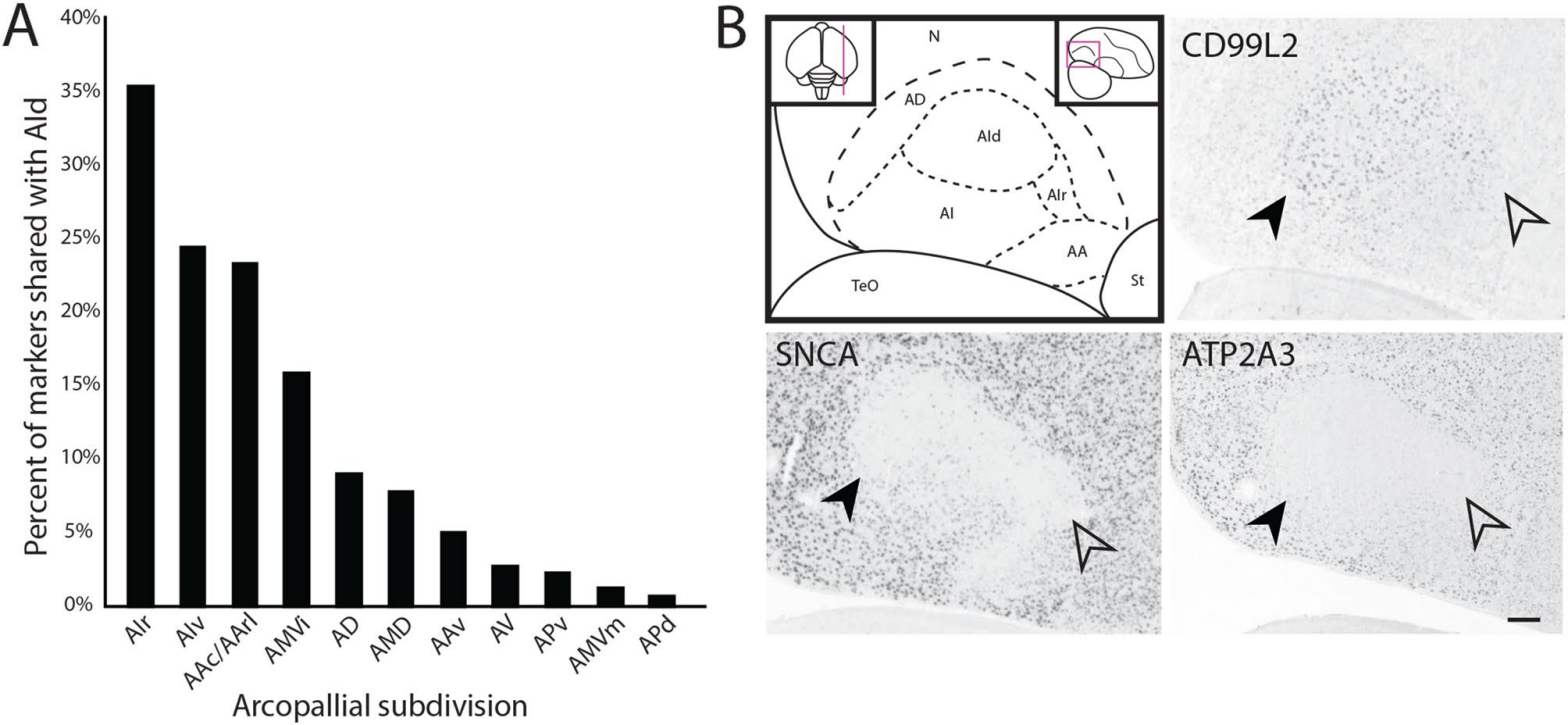
Relationship between AId and other arcopallial subdivisions. (**A**) The arcopallial expression patterns of 98 RA and AId markers were analyzed based on in situ hybridization data from ZEBrA. Plotted are the percentages of RA markers that were also considered markers of other arcopallial subdomains; individual genes can be represented in multiple columns. (**B**) Top left: drawing of arcopallium and its main subdomains on a sagittal section; top left inset indicates the position of the section (~ 2.9 mm from the midline) on a top-down view of the brain; red rectangle in the top right inset indicates the area shown in the main drawing and other panels. Other panels: In situ hybridization images of positive (*CD99L2*), negative (*ATP2A3*), and sparse cell (*SNCA*) markers of AId (black arrowheads) and AIr (empty arrowheads). Scale bar: 400 µm. *AA* anterior arcopallium, *AAc* caudal part of the anterior arcopallium, *AArl* rostro-lateral part of the anterior arcopallium, *AAv* ventral part of the anterior arcopallium, *AD* dorsal arcopallium, *AId* dorsal intermediate arcopallium, *AIr* rostral intermediate arcopallium, *AIv* ventral intermediate arcopallium, *AMVi* intermediate part of the medial ventral arcopallium, *AMD* medial dorsal arcopallium, *AMVm* medial part of the medial ventral arcopallium, *APv* ventral part of the posterior arcopallium, *APd* dorsal part of the posterior arcopallium, *AV* ventral arcopallium, *N* nidopallium, *St* striatum, *TeO* optic tectum.

We next examined whether all 162 RA markers were also differentially expressed in 12 additional molecularly defined arcopallial domains (collapsed from 19 in Mello et al.^44^). We found that > 55% of RA markers were also differential of AId, followed distantly by other domains like AIr and AA (~ 22% and ~ 18%, respectively; Fig. 4). This suggests that RA and AId are more molecularly similar to each other than to other arcopallial domains. We followed up by asking whether the 98 markers of both RA and AId were also markers of other arcopallium domains. We found that > 30% of RA/AId markers were also markers of AIr, followed distantly by AA, AMV, and AD (Fig. 5A). AIr is located rostral to and directly bordering AId, as best seen on sagittal sections (Fig. 5B, top left).

To further investigate the relationship between AId and RA, we performed a pathway enrichment analysis comparing sets of marker unique to RA with those that were both RA and AId markers, noting that a previous analysis^49^ did not consider whether RA markers were also differential in AId. For both marker sets, significantly enriched annotations related to physiological features such as regulators of cell excitability (potassium channels, voltage gated channels), regulation of intracellular calcium levels or calcium related signaling, and neuronal connectivity (summarized in Table 2; full lists in Supplemental Tables 3 and 4). While RA and AId seem to share most of their specialized molecular pathways, the sets of markers uniquely expressed in RA differentiate it from AId. We suggest that this set of genes that are unique to RA are more likely to contribute to the unique properties of RA and its role in the neurobiology of learned vocalizations (Table 1, RA unique markers).

**Table 2:** Summary of enriched pathways and related genes for RA unique and RA and AId marker sets; details in Supplemental Tables 3 and 4.

Pathway name	RA unique markers	RA and AId markers
Potassium channels	KCNA6, KCNC1, KCNF1, KCNJ6, KCNS2	KCNAB1, KCNAB2, KCNS1
Axon guidance	ARPC5, DPYSL3, PLXNA4, RELN, RHOB, SCN2A, SLIT1	CACNB2, GAP43, GRIN2B, SEMA7A, SCN1A, SCN3B, SCN4B, SCN8A, PAK6, PLXNA1, PLXNC1, ROBO1, UNC5D
GPCR binding and signaling	ACKR3, ADCYAP1, CHRM4, CRHR2, DGKZ, GRK3, HTR1B, LRP8, RHOB	ADRA1D,HRT2A, CACNB2, CRHR1, GNG2, GRIN2B, GRM3, LPL, NTSR1, PRKAR1B, RGS4, VIP

**Figure 6:**
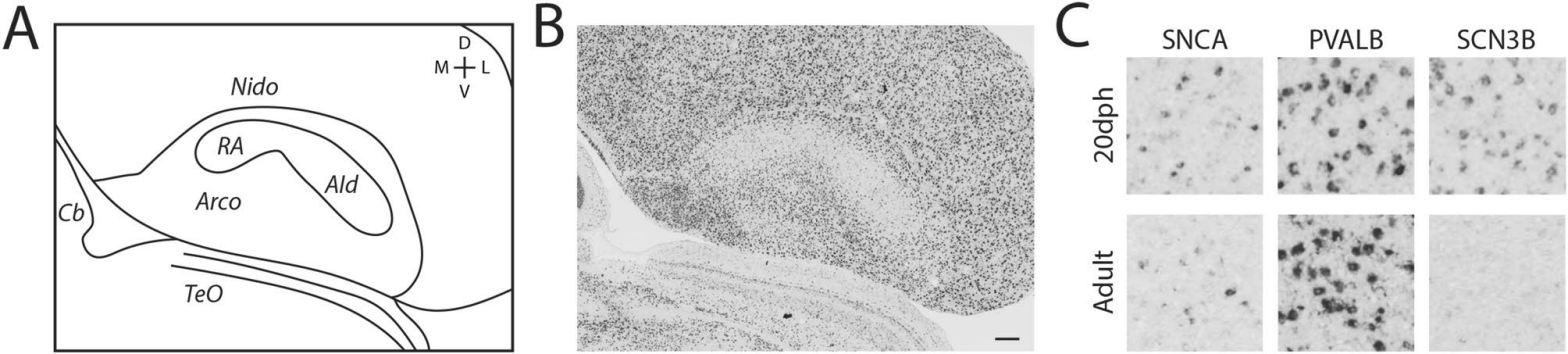
Defining AId in juvenile male zebra finch. (**A**) Drawing of the arcopallium in frontal section through the core of RA in a 20 dph male zebra finch (based on **B**), depicting the continuous area of low *SNCA* expression with sparse labeled cells that includes both RA and AId. (**B**) In situ hybridization image of *SNCA* in a 20 dph male zebra finch. (**C**) High magnification (200 × 200 µm) in situ hybridization images of AId for select adult RA and AId markers, comparing cell level expression in 20 dph juvenile and adult males. Scale bar: 200 µm. *Arco* arcopallium, *AId* dorsal intermediate arcopallium, *Cb* cerebellum, *Nido* nidopallium, *RA* robust nucleus of the arcopallium, *TeO* optic tectum.

RA in zebra finches undergoes marked developmental changes in morphology, connectivity, physiology and gene expression^61,66–70^, but except for tract-tracing data^71^, little is known with regards to age differences in AId. We therefore asked whether adult AId markers also define AId in 20 dph juvenile males entering the sensory phase of vocal learning when they can start to form an auditory memory of the tutor song^72^. They are also pre-vocal, as this age is prior to the formation of the HVC-to-RA projection, which marks the beginning of singing and of the babbling phase^61,73^. RA has also not started its massive expansion in males or regression in females^61^. *SNCA*, a robust differential marker of adult AId, was highly differential in 20 dph AId compared to surrounds, and even though RA is much smaller at this age (confirmed under Nissl), it formed a continuum of low *SNCA* expression with AId (Fig. 6A,B), noting that expression within AId was restricted to sparse cells as in adults (Fig. 6C, left). The positive marker *PVALB* showed similarly high expression in juvenile as in adult AId (Fig. 6C, middle). Thus, AId is already present in juveniles and expresses some molecular features of adult AId. In contrast, *SCN3B* showed considerable expression in juvenile AId (Fig. 6C, right) and was thus less differential compared to the adjacent arcopallium than in adults. This suggests that AId is not fully mature and undergoes further molecular differentiation until the birds reach adulthood.

**Figure 7:**
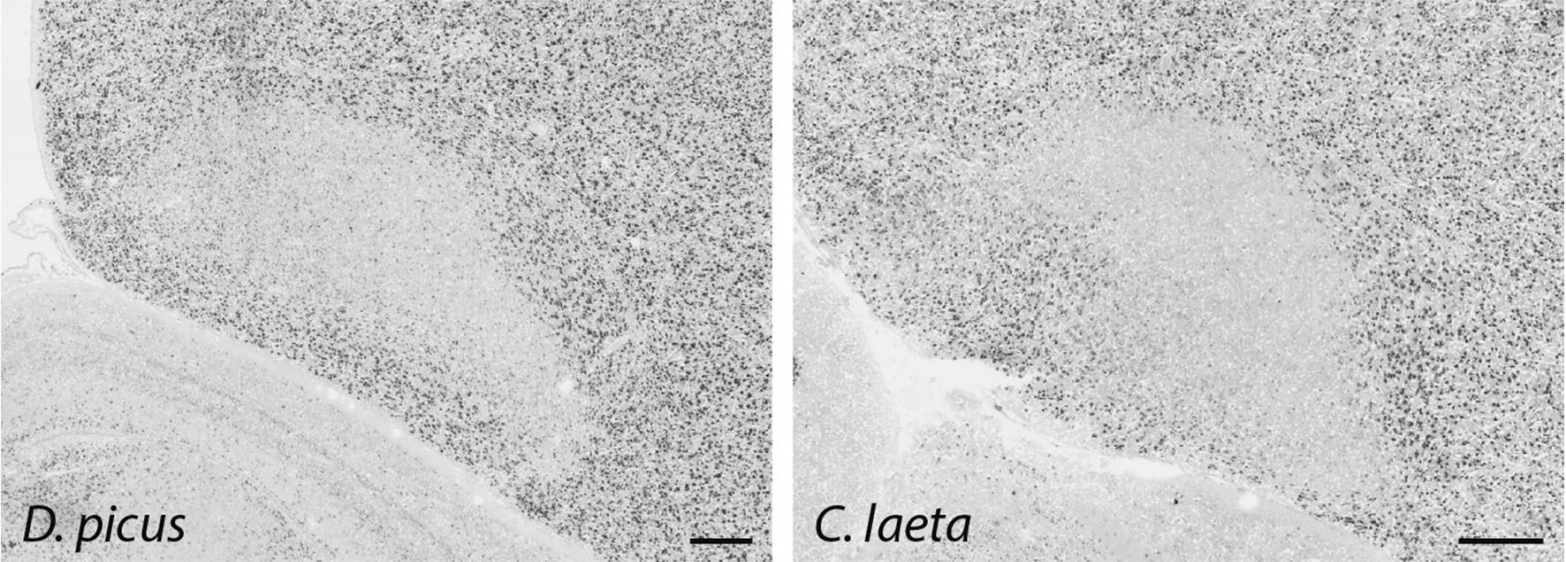
Defining AId in a suboscine. Representative in situ hybridization images of frontal sections through the arcopallium in two suboscine species, processed for an RA and AId marker (*SNCA*) from adult male zebra finches. Scale bar: 400 µm.

Previously, immediate early gene expression elicited from movement has been described in an AId-like area in vocal learning birds (songbirds, parrots, hummingbirds) and in a possibly related part of the arcopallium in a non-vocal learning avian species (doves)^33^. To further investigate if AId is present in presumed avian non-vocal learners, we next asked if molecular markers of adult AId in zebra finch also define an AId-like area in suboscine species. Suboscines, the sister taxa to the oscines (i.e. songbirds), are also passerines (perching birds) and similar to songbirds in terms of anatomy and physiology, but are generally considered to lack RA and other telencephalic vocal nuclei^16–18,47^. Differential expression of *PVALB* and androgen receptors can be interpreted as suggestive evidence of an AId-like area in the suboscine families Tyrannidae^17^ and Pipridae^74^, respectively. Here we examined males from representative species from the Thamnophilidae (Willis’s antbird; *Cercomacroides laeta*) and Dendrocolaptinae (Straight-billed woodcreeper; *Dendroplex picus*) families. In situ hybridization for the robust RA and AId marker *SNCA* in finches showed marked downregulation in a very similar area as finch AId in both suboscine species (Fig. 7), noting that an RA-like nucleus could not be identified in either species with *SNCA* or by Nissl.

## Discussion

We investigated the expression of a large set of differential arcopallial markers in zebra finches in order to better define AId in a songbird, examine the molecular relationships between RA and AId and other arcopallial domains, and more precisely identify molecular features unique to the vocal motor system. Our results provide a clear delineation of AId boundaries in adults of both sexes, and support a closer similarity between RA and AId compared to other arcopallial domains. We identify molecular specializations that are common to both RA and AId and possibly related to diverse aspects of motor control, as well as those that are unique to RA and more likely associated with vocal-motor control. We also show AId is likely present in birds that do not learn their vocalizations (non-singing females, pre-vocal juvenile males, non-vocal learner suboscines), consistent with a broader role of AId in motor control and a possible evolutionary and developmental origin of RA as a vocal-motor specialization of AId.

AId has been previously defined as a subregion of the songbird intermediate arcopallium that has distinct connectivity (discussed below) but whose boundaries are not readily identifiable under Nissl staining. Previous studies have shown that AId has distinct molecular features, including prominent expression of *PVALB* and a lack of expression of *SCN3B*^44,48,75,76^. Here we have more clearly delineated AId boundaries, and shown that it extends over a large portion of the intermediate arcopallium in both sexes. We note that given its shape and size, AId would be a difficult region to fully cover with stereotaxic injections, thus studies of connectivity need to use multiple injections over a range of coordinates to ensure full targeting, or to precisely track the injection position to address possible topography. Based on molecular similarity and spatial proximity, we also suggest that AIr might be a specialized rostral expansion of AId with a yet to be determined function.

RA and AId have several anatomical features in common, suggesting some similar functions and a close evolutionary relationship. Whereas RA receives input from HVC and from the LMAN core, the latter projection connecting the anterior forebrain and vocal-motor pathways, AId receives input from the LMAN shell and from the dorsal caudolateral nidopallium (dNCL) lateral to HVC^50,51,71,77^. The projection from dNCL to AId appears to be topographic^5,51,71,77^ suggesting that a topographic organization might also be present in a medial to lateral map in AId, with possible somatotopy. Furthermore, both RA and AId are part of the intermediate arcopallium, which originates descending somatic projections^38^ and is considered part of a general motor pathway^32^. RA projects to the medullary vocal-motor nucleus nXIIts^39^ and AId projects to targets in midbrain, pons, medulla, and possibly spinal cord, projections that could be considered analogous to the corticobulbar and corticospinal tracts in mammals^51^. Accordingly, RA and AId as well as other parts of the arcopallium (e.g. dorsal, AD) show enriched expression of markers of deep layers of the mammalian cortex^41,44^ where long descending projections originate, consistent with the idea that AId is analogous to, or contains an avian analog of deep layers of mammalian cortex^33,38,42–44,55^. Interestingly, examination of the Allen Brain Atlas mouse brain in situ hybridization data^78^ shows that some AId markers exhibit an enrichment in deep layers of the mouse motor cortex whereas others are broad deep layer cortical markers (Fig. S2).

RA and AId also seem to have analogous roles in motor control. For RA, there is evidence of severe vocal deficits after lesioning^5^ and evidence of activation during singing based on electrophysiological recordings^36,37^ and immediate early gene expression^52^. Although less studied, evidence for a motor control function for AId comes from the immediate early gene activation during movements such as wing flapping, hopping, and pecking^33,40^, and the severe motor deficits associated with large lesions^53^. Interestingly, the age differences we observed in gene expression suggest that AId undergoes molecular changes during development, which could be associated with vocal or other motor learning refinement. AId has been hypothesized to be directly involved in vocal learning^54^. We note, though, that the adjacent ventral intermediate arcopallium (AIv) is an auditory area that responds to song playbacks^79^ and may play a role in vocal learning^53,80^, thus the possible separate roles of these adjacent areas in auditory processing, vocal learning, and motor function remain to be conclusively determined.

RA and AId have previously been shown to share a few molecular markers in adult males^21,44^ but here we considerably expand that evidence, and show that AId is the arcopallial domain that shares most known markers with RA. Importantly, we have found that 25 out of the 46 genes previously identified as shared markers of songbird RA and human laryngeal motor cortex (LMC)^21^ are in fact markers of both RA and AId (Table 1). This finding argues that over half of the shared RA/LMC markers may not be uniquely associated with the vocal motor pathway or vocal control, but could perhaps subserve a broader array of somatic motor control functions. Among identified enriched pathways for these RA and AId markers is the rapid depolarization pathway containing sodium and calcium channel genes known to be differentially regulated in the song system^81^, and the axon guidance pathway containing *GAP43*, which is a shared marker of RA and LMC^21,82^. Particularly noticeable was the very low expression in RA and AId of *SNCA*, previously shown to be transcriptionally regulated in LMAN during song learning but constitutively downregulated in HVC and RA^83^. *SNCA* encodes α-synuclein, a major component of the pathological Lewy body aggregates associated with Parkinson’s disease, Lewy bodies dementia, multiple systems atrophy, and a subset of Alzheimer’s disease cases^84–86^. Aberrant transcriptional activation of *SNCA* during aging may contribute to the motor deficits seen in patients with Lewy body pathologies^87^. We suggest that downregulated *SNCA* in both vocal and presumed adjacent motor areas may serve as a protective mechanism for maintaining motor control circuits during aging in birds. Notably, we also show that a much larger set of RA markers not previously described as shared markers with LMC are also AId markers (Table 1). This includes other genes related to axonal guidance like *PLXNA1* and *ROBO1*, suggesting broader roles in motor connectivity rather than a specific role in vocal-motor circuits, as previously concluded for the latter^76^. It would be interesting to ascertain in future studies whether RA and AId markers are also differential in human primary motor cortex, using more refined dissections than those used for the human dataset in Pfenning et al.^21^.

Importantly, we have also identified molecular specializations that are unique to RA rather than common to RA and AId. These genes, which include 21 shared RA and LMC markers from Pfenning et al.^21^, now represent a better validated set of molecular features unique to this key nucleus in the circuitry for learned vocalizations. Intriguingly, many of the enriched pathways in the set of RA unique markers are similar to those of the RA and AId markers, even though the two gene lists are distinct, suggesting that specific genes within a given family may confer unique properties to each area. Axon guidance, for example, seems to be of particular relevance to the neurobiology of learned vocalizations, and axon guidance pathways have been shown to be targets for human speech and language disorders^88^. Precise vocal production relies on direct cortical projections to brainstem motor neurons that control the vocal organ, exemplified by the projection of RA to nXIIts in songbirds. Non-vocal learning animals do not have this direct projection and instead are thought to have only indirect projections from cortical motor areas to the vocal hindbrain via the midbrain^89^. A unique set of axon guidance cues likely enable the vocal-related projection from RA to the brainstem vocal nuclei to be formed and/or maintained. Some examples include *SLIT1* and *PLXNC1*, both shared markers of RA and LMC^21^ that we showed here not to be differential in finch AId, in agreement with previous observations^76^. *SLIT1* is a target of FOXP2, a transcription factor linked to speech developmental disorder^90^, and it is differentially regulated during vocal development^76^, possibly contributing to the establishment of the RA to brainstem connection. *PLXNC1* has undergone a partial duplication unique to parrots, and has been suggested as potentially linked to the expanded vocal and imitative abilities of that taxon^91^. Other axon guidance genes shown here to be differentially expressed in RA but not AId include *RELN* and *PLNXA4*, both of which also contribute to neuronal migration^92,93^, and *RTN4R* and *LINGO1*, both key components of the Nogo receptor complex and known regulators of axonal growth and myelination^94,95^.

Another pathway likely of functional relevance to RA is potassium channels, which are major determinants of neuronal excitability. Potassium channels are broadly expressed throughout the brain but song nuclei exhibit unique expression profiles of both potassium channels^96^ and other ion channel families^81^. These observations suggest that vocal nuclei are sites of differential regulation of intrinsic excitability, consistent with growing evidence that modulation of intrinsic excitable features may play important roles in regulating properties of the vocal learning circuitry^97,98^. A potassium channel gene uniquely enriched in RA is the voltage gated potassium channel subunit *KCNC1*, which encodes the K_V_3.1 protein. *KCNC1* has been associated with high-frequency firing in auditory brainstem neurons^99^, and an upregulation in RA likely contributes to the high-frequency firing capability of RA neurons^35^. We also obtained evidence that genes associated with neurotransmission and synaptic function are uniquely differential in RA, including several neurotransmitter/neuromodulatory receptors (*GABRB3*, *GABRE*, *GRK3*, *CHRM4*, *HTR1B*), in contrast to related genes also differential in AId and likely more related to synaptic regulation in the broader context of motor function (*GRIN2B*, *GRM3*, *HTR2A*). It also worth noting that some RA unique markers are transcription factors that potentially exert marked but still unexplored roles in regulating the differentiation and function of vocal circuits. This includes *SAP30*, which as part of a large histone deacetylation complex can regulate transcription and chromatin remodeling^100,101^, *NEUROD6*, which interacts with several other factors (*TBR1, FEZF2, FOXG1, SATB2*, *EMX1*) linked to cortical development^102^ and is involved in regulation of callosal projections^103^, and *RORA*, which has been implicated in cortical and cerebellar development^104^ and autism^105^.

We note that RA in both juvenile males and adult females, while smaller than in adult males, is still continuous with the medial end of AId. It thus appears that as RA undergoes its developmental growth in males, it likely expands medially and ventrally so that in adults it ends closely adjacent to the medial arcopallium (AM) and to the RA cup, the latter considered part of AIv and related to auditory processing^29,31,106^. However, RA is very distinct from AM and AIv, both molecularly and in terms of connectivity^31,44,54,107^. Our observations support a much closer relationship between RA and AId. It is not known, however, if AId has unique molecular features, as all known markers of AId are genes initially identified as RA markers. Furthermore, the evidence for molecular similarities between AId in songbirds and in non-vocal learning suboscines is consistent with a broader motor function for AId, as contrasted to the exceptional specialization of RA for vocal-motor function. It also supports the notion that RA may have evolved as a specialization of a primordial motor region present in birds independently of the occurrence of learned vocalizations, referred to as the motor theory for vocal learning origin^33^, favoring it over an auditory origin^30,31^. It is important to note that under this hypothesis, RA and AId would not have to be the sole motor output of the zebra finch arcopallium. The AD, located directly dorsal to RA and AId^44^ also has somatic-like projections to the thalamus, midbrain, and brainstem^38^ and may also represent an avian analog to layers 5/6 of motor cortical areas. While several arcopallial domains express markers of both mammalian cortical and amygdalar subdivisions, AD predominantly expresses markers of layer 6 cortical neurons^44^. It is unknown if neurons analogous to pyramidal projection neurons in layers 5/6 are intermixed throughout the arcopallium or segregated into separate domains.

In summary, we provide molecular evidence for a close relationship between RA and AId, as well as clearly identify molecular specializations unique to RA. Our findings are consistent with AId being an ancestral motor region from which the vocal nucleus RA may have evolved. The data also provide an invaluable source of candidate genes for future studies on specialized vocal learning mechanisms.

## Supplementary information

Supplementary Information 1.

Supplementary Information 2.

Supplementary Information 3.

Supplementary Information 4.

Supplementary Information 5.

## References

[1] Zeigler HP, Marler P, Zeigler HP, Marler P (2004). Behavioral Neurobiology of Birdsong.

[2] Reiner A (2004). Revised nomenclature for avian telencephalon and some related brainstem nuclei. J. Comp. Neurol..

[3] Jarvis ED (2005). Avian brains and a new understanding of vertebrate brain evolution. Nat. Rev. Neurosci..

[4] Lovell PV (2020). ZEBrA: Zebra finch expression brain atlas—A resource for comparative molecular neuroanatomy and brain evolution studies. J. Comp. Neurol..

[5] Nottebohm F, Stokes TM, Leonard CM (1976). Central control of song in the canary, *Seinus canarius*. J. Comp. Neurol..

[6] Bottjer SW, Halsema KA, Brown SA, Miesner EA (1989). Axonal connections of a forebrain nucleus involved with vocal learning in zebra finches. J. Comp. Neurol..

[7] Brainard MS, Doupe AJ (2002). What songbirds teach us about learning. Nature.

[8] Mello CV (2014). The zebra finch, *Taeniopygia guttata*: An avian model for investigating the neurobiological basis of vocal learning. Cold Spring Harbor Protocols..

[9] Paton JA, Manogue KR, Nottebohm F (1981). Bilateral organization of the vocal control pathway in the budgerigar, Melopsittacus undulatus. J. Neurosci..

[10] Durand SE, Heaton JT, Amateau SK, Brauth SE (1997). Vocal control pathways through the anterior forebrain of a parrot (*Melopsittacus undulatus*). J. Comp. Neurol..

[11] Jarvis ED, Mello CV (2000). Molecular mapping of brain areas involved in parrot vocal communication. J. Comp. Neurol..

[12] Jarvis ED (2000). Behaviourally driven gene expression reveals song nuclei in hummingbird brain. Nature.

[13] Gahr M (2000). Neural song control system of hummingbirds: comparison to swifts, vocal learning (Songbirds) and nonlearning (Suboscines) passerines, and vocal learning (Budgerigars) and nonlearning (Dove, owl, gull, quail, chicken) nonpasserines. J. Comp. Neurol..

[14] Karten HJ, Hodos W (1967). A stereotaxic atlas of the brain of the pigeon (Columba livia).

[15] Kuenzel WJ, Masson M (1988). A stereotaxic atlas of the brain of the chick (Gallus domesticus).

[16] Kroodsma DE, Konishi M (1991). A suboscine bird (eastern phoebe, *Sayornis phoebe*) develops normal song without auditory feedback. Anim. Behav..

[17] Liu WC, Wada K, Jarvis ED, Nottebohm F (2013). Rudimentary substrates for vocal learning in a suboscine. Nat. Commun..

[18] de Lima JL (2015). A putative RA-like region in the brain of the scale-backed antbird, *Willisornis peocilinotus*, (Furnariides, Suboscines, Passeriformes, Thamnophilidae). Genet. Mol. Biol..

[19] Doupe AJ, Kuhl PK (1999). Birdsong and human speech: Common themes and mechanisms. Annu. Rev. Neurosci..

[20] Wirthlin M (2019). A modular approach to vocal learning: Disentangling the diversity of a complex behavioral trait. Neuron.

[21] Pfenning AR (2014). Convergent transcriptional specializations in the brains of humans and song-learning birds. Science.

[22] Knörnschild M (2014). Vocal production learning in bats. Curr. Opin. Neurobiol..

[23] Janik VM (2014). Cetacean vocal learning and communication. Curr. Opin. Neurobiol..

[24] Jurgens U (1998). Neuronal control of mammalian vocalization, with special reference to the squirrel monkey. Naturwissenschaften.

[25] Hammerschmidt K, Jürgens U, Freudenstein T (2001). Vocal development in squirrel monkeys. Behvavior..

[26] Hammerschmidt K (2012). Mice do not require auditory input for the normal development of their ultrasonic vocalizations. BMC Neurosci..

[27] Arriaga G, Zhou EP, Jarvis ED (2012). Of mice, birds, and men: The mouse ultrasonic song system has some features similar to humans and song-learning birds. PLoS ONE.

[28] Mahrt EJ, Perkel DJ, Tong L, Rubel EW, Portfors CV (2013). Engineered deafness reveals that mouse courtship vocalizations do not require auditory experience. J. Neurosci..

[29] Kelly DB, Nottebohm F (1979). Projections of a telencephalic auditory nucleus-field L-in the canary. J. Comp. Neurol..

[30] Margoliash D (1994). Distributed representation in the song system of oscines: Evolutionary implications and functional consequences. Brain Behav. Evol..

[31] Mello CV, Vates GE, Okuhata S, Nottebohm F (1998). Descending auditory pathways in the adult male zebra finch (*Taeniopygia guttata*). J. Comp. Neurol..

[32] Farries MA (2004). The avian song system in comparative perspective. Ann. N. Y. Acad. Sci..

[33] Feenders G (2008). Molecular mapping of movement-associated areas in the avian brain: A motor theory for vocal learning origin. PLoS ONE.

[34] Jarvis ED (2019). Evolution of vocal learning and spoken language. Science.

[35] Yu AC, Margoliash D (1996). Temporal hierarchical control of singing in birds. Science.

[36] Hahnloser RH, Kozhevnikov AA, Fee MS (2002). An ultra-sparse code underlies the generation of neural sequences in a songbird. Nature.

[37] Leonardo A, Fee MS (2005). Ensemble coding of vocal control in birdsong. J. Neurosci..

[38] Zeier H, Karten HJ (1971). The archistriatum of the pigeon: Organization of afferent and efferent connections. Brain Res..

[39] Wild JM (1993). Descending projections of the songbird nucleus robustus archistriatalis. J. Comp. Neurol..

[40] Yuan RC, Bottjer SW (2020). Multi-dimensional tuning in motor cortical neurons during active behavior. eNeuro..

[41] Dugas-Ford J, Rowell JJ, Ragsdale CW (2012). Cell-type homologies and the origins of the neocortex. Proc. Natl. Acad. Sci. U.S.A..

[42] Jarvis ED (2013). Global view of the functional molecular organization of the avian cerebrum: Mirror images and functional columns. J. Comp. Neurol..

[43] Herold C, Paulitschek C, Palomero-Gallagher N, Güntürkün O, Zilles K (2018). Transmitter receptors reveal segregation of the arcopallium/amygdala complex in pigeons (*Columba livia*). J. Comp. Neurol..

[44] Mello CV, Kaser T, Buckner AA, Wirthlin M, Lovell PV (2019). Molecular architecture of the zebra finch arcopallium. J. Comp. Neurol..

[45] Martínez-García F, Marínez-Marcos A, Lanuza E (2002). The pallial amygdala of amniote vetebrates: Evolution of the concept, evolution of the structure. Brain Res. Bull..

[46] Vicario A, Mendoza E, Abellán A, Scharff C, Medina L (2017). Genoarchitecture of the extended amygdala in zebra finch, and expression of FoxP2 in cell corridors of different genetic profile. Brain Struct. Funct..

[47] Saldanha C, Schultz JD, London SE, Schlinger BA (2000). Telencephalic aromatase but not a song circuit in a sub-oscine passerine, the golden collared manakin (*Manacus vitellinus*). Brain Behav. Evol..

[48] Hara E, Rivas MV, Ward JM, Okanoya K, Jarvis ED (2012). Convergent differential regulation of parvalbumin in the brains of vocal learners. PLoS ONE.

[49] Lovell PV, Huizinga NA, Friedrich SR, Wirthlin M, Mello CV (2018). The constitutive differential transcriptome of a brain circuit for vocal learning. BMC Genom..

[50] Johnson F, Sablan MM, Bottjer SW (1995). Topographic organization of a forebrain pathway involved with vocal learning in zebra finches. J. Comp. Neurol..

[51] Bottjer SW, Brady JD, Cribbs B (2000). Connections of a motor cortical region in zebra finches: Relation to pathways for vocal learning. J. Comp. Neurol..

[52] Jarvis ED, Nottebohm F (1997). Motor-driven gene expression. Proc. Natl. Acad. Sci. U.S.A..

[53] Mandelblat-Cerf Y, Las L, Denisenko N, Fee MS (2014). A role for descending auditory cortical projections in songbird vocal learning. Elife..

[54] Bottjer SW, Altenau B (2010). Parallel pathways for vocal learning in basal ganglia of songbirds. Nat. Neurosci..

[55] Jarvis ED (2004). Learned birdsong and the neurobiology of human language. Ann. N. Y. Acad. Sci..

[56] Replogle K (2008). The Songbird Neurogenomics (SoNG): Initiative: community-based tools and strategies for study of brain gene function and evolution. BMC Genom..

[57] Carleton JB (2014). An optimized protocol for high-throughput in situ hybridization of zebra finch brain. Cold Spring Harb Protoc..

[58] Schindelin J (2012). Fiji: An open-source platform for biological-image analysis. Nat. Methods..

[59] Kamburov A, Stelzl U, Lehrach H, Herwig R (2013). The ConsensusPathDB interaction database: 2013 update. Nucleic Acids Res..

[60] Lovell PV (2018). Curation of microarray oligonucleotides and corresponding ESTs/cDNAs used for gene expression analysis in zebra finches. BMC Res. Notes..

[61] Konishi M, Akutagawa E (1985). Neuronal growth, atrophy and death in a sexually dimorphic song nucleus in the zebra finch brain. Nature.

[62] Spiro JE, Dalva MB, Mooney R (1999). Long-range inhibition within the zebra finch song nucleus RA can coordinate the firing of multiple projection neurons. J. Neurophyiol..

[63] Nottebohm F, Arnold AP (1976). Sexual dimorphism in vocal control areas of the songbird brain. Science.

[64] Nixdorf-Bergweiler BE (1996). Divergent and parallel development in volume sizes of telencephalic song nuclei in male and female zebra finches. J. Comp. Neurol..

[65] Karten HJ (2013). Digital atlas of the zebra finch (*Taeniopygia guttata*) brain: A high-resolution photo atlas. J. Comp. Neurol..

[66] Bottjer SW, Miesner EA, Arnold AP (1986). Changes in neuronal number, density and size account for increases in volume of song-control nuclei during song development in zebra finches. Neurosci. Lett..

[67] Ölveczky BP, Otchy TM, Goldberg JH, Aronov D, Fee MS (2011). Changes in the neural control of a complex motor sequence during learning. J Neurophysiol..

[68] Tang YP, Wade J (2013). Developmental changes in BDNF protein in the song control nuclei of zebra finches. Neuroscience.

[69] Merullo DP (2018). Neurotensin and neurotensin receptor 1 mRNA expression in song-control regions changes during development in male zebra finches. Dev. Neurobiol..

[70] Hayase S (2018). Vocal practice regulates singing activity-dependent genes underlying age-independent vocal learning in songbirds. PLoS Biol..

[71] Iyengar S, Viswanathan SS, Bottjer SW (1999). Development of topography within song control circuitry of zebra finches during the sensitive period for song learning. J. Neurosci..

[72] Eales LA (1985). Song learning in zebra finches: some effects of song model availability on what is learnt and when. Anim. Behav..

[73] Aronov D, Andalman AS, Fee MS (2008). A specialized forebrain circuit for vocal babbling in the juvenile songbird. Science.

[74] Fusani L, Donaldson Z, London SE, Fuxjager MJ, Schlinger BA (2014). Expression of androgen receptor in the brain of a sub-oscine bird with an elaborate courtship display. Neurosci. Lett..

[75] Riters LV, Ball GF (2002). Sex differences in the densities of alpha 2-adrenergic receptors in the song control system, but not the medial preoptic nucleus in zebra finches. J. Chem. Neuroanat..

[76] Wang R (2015). Convergent differential regulation of SLIT-ROBO axon guidance genes in the brains of vocal learners. J. Comp. Neurol..

[77] Fernández M (2020). Parallel organization of the avian sensorimotor arcopallium: Tectofugal visual pathway in the pigeon (*Columba livia*). J. Comp. Neurol..

[78] Lein ES (2007). Genome-wide atlas of gene expression in the adult mouse brain. Nature.

[79] Yuan RC, Bottjer SW (2019). Differential developmental changes in cortical representations of auditory-vocal stimuli in songbirds. J. Neurophysiol..

[80] Kearney MG, Warren TL, Hisey E, Qi J, Mooney R (2019). Discrete evaluative and premotor circuits enable vocal learning in songbirds. Neuron.

[81] Friedrich SR, Lovell PV, Kaser TM, Mello CV (2019). Exploring the molecular basis of neuronal excitability in a vocal learner. BMC Genom..

[82] Clayton DF, George JM, Mello CV, Siepka SA (2009). Conservation and expression of IQ-domain-containing calpacitin gene products (Neuromodulin/GAP-43, Neurogranin/PC3) in the adult and developing oscine song control system. Dev. Neurobiol..

[83] George JM, Jin H, Woods WS, Clayton DF (1995). Characterization of a novel protein regulated during the critical period for song learning in the zebra finch. Neuron.

[84] Spillantini MG, Schmidt ML, Lee VM, Trojanowski JQ, Jakes R, Goedert M (1997). Alpha-synuclein in Lewy bodies. Nature.

[85] Singleton AB (2003). Alpha-Synuclein locus triplication causes Parkinson’s disease. Science.

[86] Masliah E (2001). Beta-amyloid peptides enhance alpha-synuclein accumulation and neuronal deficits in a transgenic mouse model linking Alzheimer’s disease and Parkinson’s disease. Proc. Natl. Acad. Sci. U.S.A..

[87] Scherzer CR (2008). GATA transcription factors directly regulate the Parkinson’s disease-linked gene alpha-synuclein. Proc. Natl. Acad. Sci. U.S.A..

[88] Lei H (2017). Axon guidance pathways served as common targets for human speech/language evolution and related disorders. Brain Lang..

[89] Petkov CI, Jarvis ED (2012). Birds, primates, and spoken language origins: Behavioral phenotypes and neurobiological substrates. Front. Evol. Neurosci..

[90] Sin C, Li H, Crawford DA (2015). Transcriptional regulation by FOXP1, FOXP2, and FOXP4 dimerization. J. Mol. Neurosci..

[91] Wirthlin M (2018). Parrot genomes and the evolution of heightened longevity and cognition. Curr. Biol..

[92] de Lambert RC, Goffinet AM (1998). The reeler mouse as a model of brain development. Adv. Anat. Embryol. Cell Biol..

[93] Winberg ML (1998). Plexin A is a neuronal semaphorin receptor that controls axon guidance. Cell.

[94] Mi S (2004). LINGO-1 is a component of the Nogo-66 receptor/p75 signaling complex. Nat. Neurosci..

[95] Fournier AE, GrandPre T, Strittmatter SM (2001). Identification of a receptor mediating Nogo-66 inhibition of axonal regeneration. Nature.

[96] Lovell PV, Carleton JB, Mello CV (2013). Genomics analysis of potassium channel genes in songbirds reveals molecular specializations of brain circuits for the maintenance and production of learned vocalizations. BMC Genom..

[97] Ross MT, Flores D, Bertram R, Johnson F, Hyson RL (2017). Neuronal intrinsic physiology changes during development of a learned behavior. eNeuro..

[98] Daou A, Margoliash D (2020). Intrinsic neuronal properties represent song and error in zebra finch vocal learning. Nat. Commun..

[99] Wang LY, Gan L, Forsynthe ID, Kaczmarek LK (1998). Contribution of the Kv3.1 potassium channel to high-frequency firing in mouse auditory neurones. J. Physiol..

[100] Viiri KM (2009). DNA-binding and -bending activities of SAP30L and SAP30 are mediated by a zinc-dependent module of monophosphoinositides. Mol. Cell. Biol..

[101] Xie T (2011). Structure of the 30-kDa Sin3-associated protein (SAP30) in complex with the mammalian Sin3A corepressor and its role in nucleic acid binding. J. Biol. Chem..

[102] Kang HJ (2011). Spatiotemporal transcriptome of the human brain. Nature.

[103] Bormuth I (2013). Neuronal basic helix-loop-helix proteins Neurod2/6 regulate cortical commissure formation before midline interactions. J. Neurosci..

[104] Nakagawa Y, O’Leary DDM (2003). Dynamic patterned expression of orphan nuclear receptor genes RORalpha and RORbeta in developing mouse forebrain. Dev. Neurosci..

[105] Sarachana T, Hu VW (2013). Genome-wide identification of transcriptional targets of RORA reveals direct regulation of multiple genes associated with autism spectrum disorder. Mol. Autism..

[106] Vates GE, Broome BM, Mello CV, Nottebohm F (1996). Auditory pathways of caudal telencephalon and their relation to the song system of adult male zebra finches. J. Comp. Neurol..

[107] Cheng M-F, Chaiken M, Zuo M, Miller H (1999). Nucleus taenia of the amygdala of birds: anatomical and functional studies in Ring Doves (*Streptopelia risoria)* and European Starlings (*Sturnus vulgaris*). Brain Behav. Evol..

