# Supplementary figures and images for: Molecular specializations of deep cortical layer analogs in songbirds
Source: Sci Rep. 2020 Oct 30;10:18767. doi: 10.1038/s41598-020-75773-4 (PMC7599217; doi:10.1038/s41598-020-75773-4)

Supplemental Figure 1

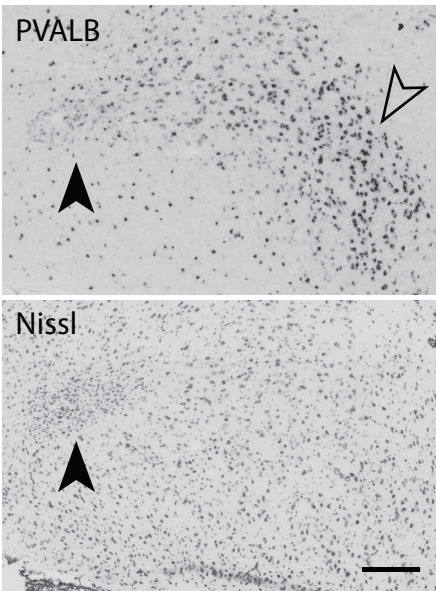

Supplemental Figure 2

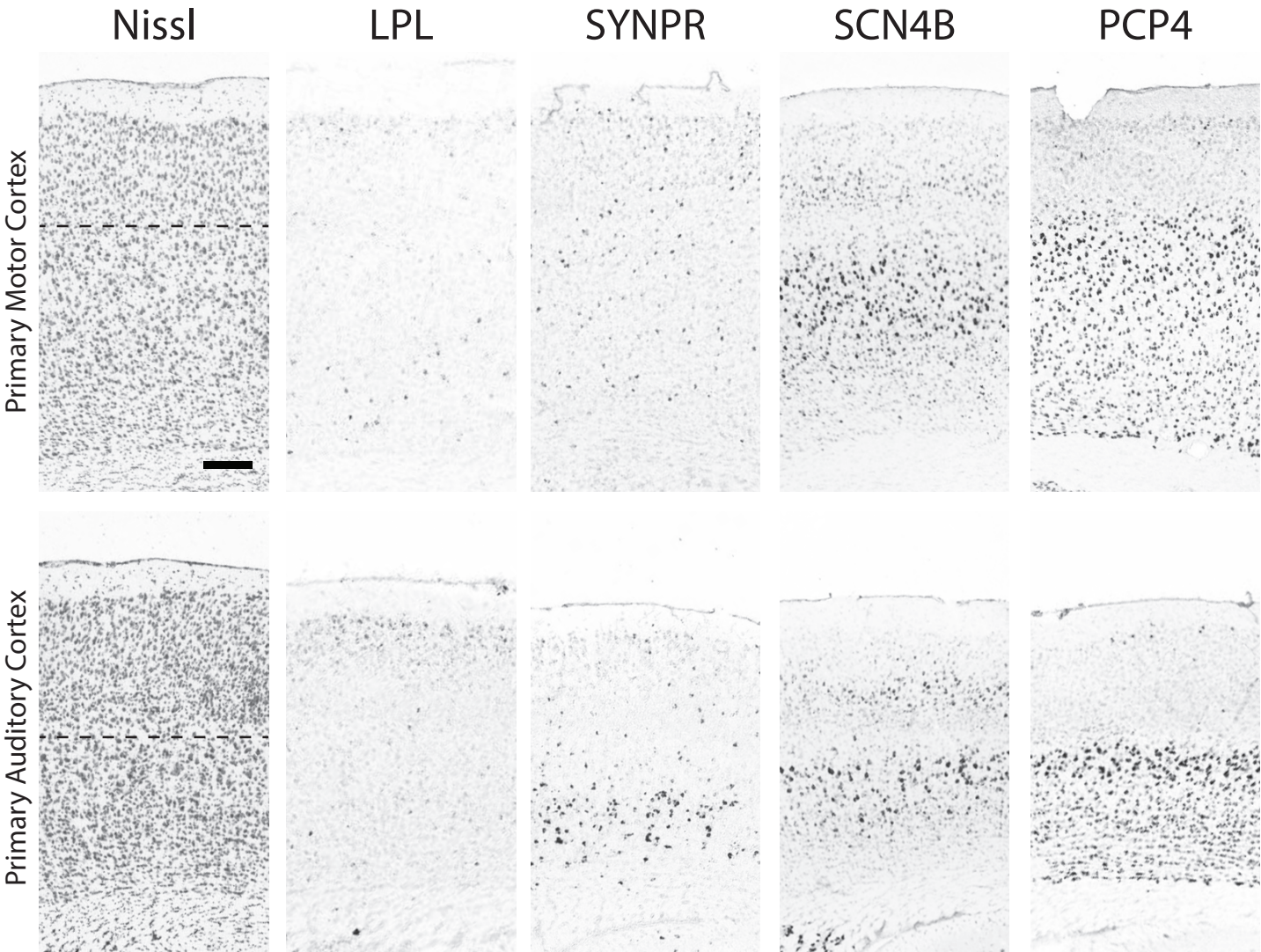

Supplement: Supplementary file 1 — Supplementary Information 1. [file 41598_2020_75773_MOESM1_ESM.pdf]
